# Fusing Ambient and Mobile Sensor Features Into a Behaviorome for Predicting Clinical Health Scores

**DOI:** 10.1109/access.2021.3076362

**Published:** 2021-04-28

**Authors:** DIANE J. COOK, MAUREEN SCHMITTER-EDGECOMBE

**Affiliations:** 1 School of Electrical Engineering and Computer Science, Washington State University, Pullman, WA 99164, USA; 2 Department of Psychology, Washington State University, Pullman, WA 99164, USA

**Keywords:** Activity recognition, behavior markers, joint inference, smart homes, smartwatches

## Abstract

Advances in machine learning and low-cost, ubiquitous sensors offer a practical method for understanding the predictive relationship between behavior and health. In this study, we analyze this relationship by building a behaviorome, or set of digital behavior markers, from a fusion of data collected from ambient and wearable sensors. We then use the behaviorome to predict clinical scores for a sample of n = 21 participants based on continuous data collected from smart homes and smartwatches and automatically labeled with corresponding activity and location types. To further investigate the relationship between domains, including participant demographics, self-report and external observation-based health scores, and behavior markers, we propose a joint inference technique that improves predictive performance for these types of high-dimensional spaces. For our participant sample, we observe correlations ranging from small to large for the clinical scores. We also observe an improvement in predictive performance when multiple sensor modalities are used and when joint inference is employed.

## I. INTRODUCTION

Care providers, patients, families, and researchers all struggle to understand human health and its influences. Until recently, theories that connect behavior and health were based on self-report, which can suffer from retrospective memory limitations [1], or experimenter observations, which can introduce confounds and unintended bias [2]. Because we can now collect continuous behavior and health data in an ecologically-valid manner, we can leverage substantial evidence to support or contradict these theories. Despite the tremendous potential of emerging technologies to monitor and assess health and behavior, many of them are constrained to operate in controlled environments, laboratory settings, and clinics.

We hypothesize that multi-modal sensor data collected in uncontrolled settings can be harnessed to predictively connect health and behavior. Such time series data consist of sensor state information along with a timestamp for when the state change occurred. Using activity recognition and machine learning, we will investigate whether clinical scores can be predicted from time series data. If successful, such predictions can provide unprecedented, naturalistic insight into a person’s health status and the relationship between behavior and health. They will allow caregivers to track and predict health trajectories over time, which may be particularly valuable for individuals going through rehabilitation or experiencing cognitive decline. Sensor-based continual assessment of daily physical and behavior patterns, combined with predicted clinical scores, provides insights that cannot be gleaned from spending a few minutes in a doctor’s office or clinic.

The specific research contributions we make in this paper are the following. First, we describe methods of labeling ambient sensor and wearable sensor data in real time with corresponding activity labels. Second, we introduce methods to extract a behaviorome, or set of digital behavior markers, from activity-labeled time series data. Third, we investigate the predictability of clinical scores from a person’s behaviorome. In this investigation, we collect continuous smart home and smartwatch data for n = 21 healthy older adults while they perform their normal daily routines. Finally, we introduce a method to enhance clinical score predictability by leveraging the relationships between multiple target variables.

## II. RELATED WORK

Sensor data hold the potential to give researchers substantial information about a person’s behavior as well as health. While sensors have become low cost, wireless, and deployable in real-world settings, researchers have found no single “silver bullet” sensor that provides all the necessary insight into a person’s health and behavior. However, researchers have made progress in linking features of individual sensor modalities with specific health characteristics. Here we review recent advances in analyzing time series sensor data to gain insights for automated health assessment.

Our study combines behavior markers from wearable sensors and ambient sensors. Other sensors could be considered for this task as well, such as video, audio, phone, and biometric, to name a few. We restrict our analysis to sensors which are commercially available, easily deployable, and able to handle a variety of environment conditions. Within these constraints, researchers have made considerable progress in performing health assessment from sensor data. One such sensor platform is provided by wearable and mobile sensors. Wearable sensors typically quantify aspects of movement. These sensors may be embedded into inertial measurement units (IMUs) or included on commercial phones and watches which collect acceleration and angular velocity data.

In one study, Mancini *et al.* [3] analyzed wearable data to analyze movement parameters when a person performed turning motions. Customized wearable devices collected angular velocities. These velocities were shown to be predictive of falls and of cognitive function as determined by Clinical Dementia Rating scores. In other work, Adler *et al.* [4] collected data from a commercial smartphone to predict schizophrenia-related psychotic relapse using a neural network. They extracted features from movement-based sensors but also included app use, phone calls, and inferred sleep times. In addition to these features, Wang *et al.* [5] also inferred movement category (e.g., walking, in a vehicle) and computed an index that estimates the regularity of a person’s daily schedule from phone-based sensors. They used this information to infer symptoms of depression as indicated by in-the-moment Patient Health Questionnaire-4 (PHQ-4). Huckins *et al.* [6] utilized a similar set of features to predict PHQ-4-detected anxiety among college students at the beginning of the COVID-19 pandemic. Focusing on a different health domain, Newland *et al.* [7] analyzed movement data collected from wearable sensors to infer clinical scores that are specific for assessing symptoms of multiple sclerosis.

In other studies, researchers utilized information from wearable sensors to infer a behavior or health marker that is consistent with a larger health condition. For example, Fiorini *et al.* [8] analyzed statistical features from wearable accelerometer and angular velocity readings to measure a person’s auditory sustained attention. This measure, in turn, provided insights on the person’s cognitive function and thus the impact of cognitive training on cognitive function. Fallahzadeh *et al.* [9] collected movement and location information on smartphones to predict and promote medication adherence. They extracted statistical features from movement-based sensors, labeled data with corresponding activities categories, and used this information to infer whether a patient would be adherent to their medication schedule at the prescribed time based on their activity context.

Wearable sensors are particularly insightful at analyzing and inferring movement-based parameters that help to determine a person’s health status. In particular, Ravichandran *et al.* [10] surveyed and compared the effectiveness of alternative commercial accelerometer-based devices for detecting sleep stages and durations. Fallmann and Chen [11] also surveyed approaches to detect and analyze sleep stages from wearable sensor data, focusing on research advances. Lee *et al.* [12] analyzed smartphone motion sensors to measure walking movements of healthy individuals and an individual with back pain. In particular, they calculated the variation of rotation about three axes and found that the participant with back pain demonstrated a wider range of variations, indicating a diminished level of walking balance.

The above studies primarily analyze movement sensors to provide insights on health status and behavior parameters that impact health status. However, some recent work provides evidence that location information can also be gathered from mobile devices and generalized among individuals. Lin and Hsu [13] introduced multiple generalizable location features, including heading change rate, distance covered, and velocity change rate. They also suggested clustering location readings to detect person-specific frequented spots. Boukhechba *et al.* [14] employed this strategy to identify significant locations. They used this information together with phone call frequency to predict a person’s social anxiety.

Similar to these reviewed studies, we extract features from both movement and location information provided by wearable devices. Wearable devices offer other data modalities as well. These include phone usage, heart rate, and even audio and video data. These data sources offer valuable behavior and health features. For example, Tseng *et al.* [15] found that they could predict a person’s inhibitory control based solely on phone usage statistics. To enable longitudinal data collection without additional hardware and participant involvement, we do not incorporate these additional wearable information sources into the analysis.

Ambient sensors that are embedded into physical environments complement wearable sensors by localizing a person within the building. This information, combined with environment state readings, can be used to longitudinally track complex activity patterns. As in the case of analyzing wearable sensors, researchers have devised methods to extract features from longitudinal ambient sensor data that provide insights on behavior patterns. For example, Austin *et al.* [16] inferred behavior parameters from passive infrared motion sensors. The parameters include time spent out of the home, indoor walking speed, and time spent sleeping. From these features, they were able to predict loneliness among older adults. Akl *et al.* [17] also analyzed ambient motion sensor data to calculate times that individuals spent in rooms of the house. Based on this information, they were able to infer a person’s cognitive health as indicated by Mini-Mental State Examination and Clinical Dementia Rating scores. Dawadi *et al.* [18] similarly extracted features from motion and door sensors. From these features, they inferred activity categories and mapped activity features (duration and time of day) onto clinical cognitive health indicators including the Repeatable Battery of Neuropsychological Status and Timed Up and Go scores. Aramendi *et al.* [19] collected similar activity features over multiple years to predict these scores as well as scores on a self-report measure of instrumental activities of daily living (IADL-C).

Fritz *et al.* [20] collected similar features from ambient sensors as the previous studies. In this case, however, the features were used to detect pain episodes for participants with chronic health conditions. Skubic *et al.* [21] also collected data in naturalistic settings to identify possible health events. Unlike the previous studies, they analyzed video data together with ambient sensors, and created rules to identify anomalies from extracted features that increased the possibility of detecting events requiring intervention. In our study, we include features collected from motion, door, light, and temperature ambient sensors. As indicated by these prior studies, such sensors can be used to recognize activities and in turn provide insights on health status.

In all of these prior works, as in this current study, the goal of the technology is monitoring behavior over time, in the wild, to perform health assessment. In contrast to prior work, this study extracts a large set of behavior markers from both wearable and ambient sensor data. Because sensor platforms provide distinct insights in these routines, we hypothesize that the fused information will provide a more comprehensive picture of behavior patterns and thus improve predictive performance over a range of clinical health scores. Additionally, we introduce a method to predict multiple health scores in a way that boosts the prediction of each health parameter. These insights can be used to better understand the impact of behavior on health and to inform the continued design of assessment measures.

## III. METHODS

### A. DATA COLLECTION

**FIGURE 1.**
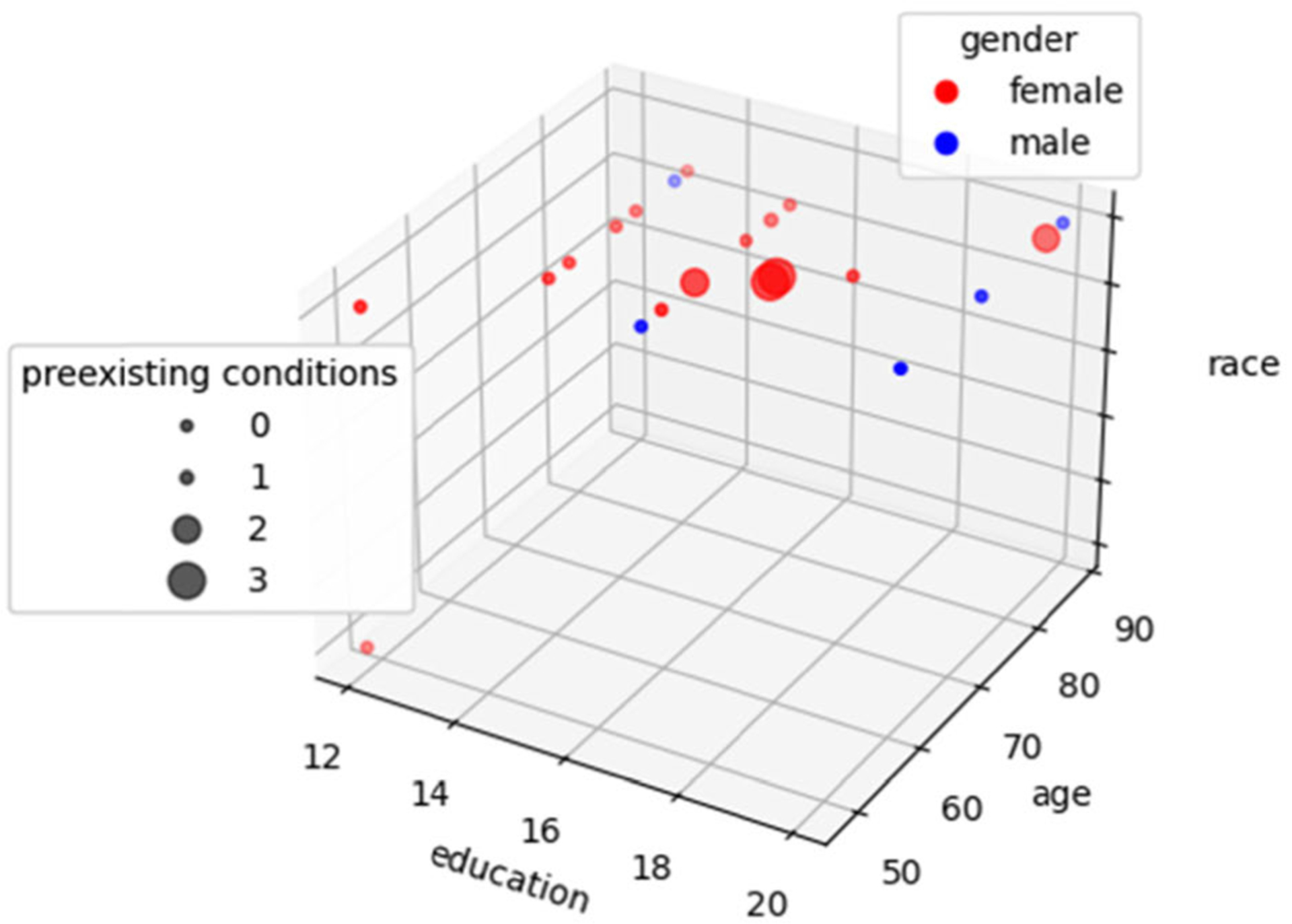
Distribution of study participants based on years of education, race, age, gender, and the number of preexisting conditions in these categories: neurological disability, head injury, and experiencing changes in memory performance.

In this paper, we analyze smart home data collected from 21 healthy older adult volunteers (16 female, 5 male). The mean age was 69 (SD 10.9) and the distribution of demographics is shown in Fig. 1. All participants were recruited through community advertisements or referral from physicians and local community agencies. Before they were selected for the study, participants completed a health screening interview and the Telephone Interview of Cognitive Status [22] over the phone. The interview allowed experimenters to rule out exclusion criteria. In particular, we enrolled participants who were at least 45 years of age and did not indicate a possibility of dementia or lack of insight that could lead to unreliable self-report data (TICS ≥ 26). The data were collected as part of a larger study focused on developing and validating methods for health assessment and intervention that utilize continuously-collected sensor data in naturalistic, real-world settings. The parent study does not target specific distributions of education levels, age, gender, or race.

We equipped the homes of these study participants with a CASAS “smart home in a box” [23]. Each CASAS smart home apartment contains ambient sensors, consisting of passive infrared motion/light sensors attached to the ceilings and door/temperature sensors attached to external doors and commonly-used cabinets. In participant homes, experimenters install approximately two sensors per room with adhesive strips that can be removed at the end of data collection. Data can be collected from these sensors for long periods of time while requiring no extra tasks from the participants, and the efficacy of these sensors to capture activity patterns and indicate health status has been demonstrated in prior work [19], [24], [25]. The CASAS sensors generate timestamped readings whenever the sensed state changes (e.g., still to motion, door closed to open). We collected 1 month of ambient sensor data for each participant. A 1-month data collection period was chosen to ensure that routine behavior patterns that occur on a daily, weekly, or monthly basis would be observed. This study was approved by the Washington State University Institutional Review Board.

During the same month, we also collected continuous smartwatch data for each participant. Participants wore the watch continuously while data were collected at 1Hz. To provide continuous data collection, participants wore one watch during the day while charging a second watch, then wore the other watch at night while charging the first. This sample rate was selected to ensure that data could be collected continuously during the day without draining the battery. Consistent with prior studies [3]–[8], smartwatch data consist of timestamped readings for 3D accelerometer, 3D gyroscope, course, and speed. We additionally collected location data. As suggested in earlier work [13], [14], raw location coordinates were not included in the models because of the privacy risk and because they do not generalize well to new participants. Instead, we employ an open street map to map each coordinate onto a location type (home, road, work, other).

Prior activity recognition and health assessment research offers evidence that movement-based sensors including accelerometers and gyroscopes provide insight on behavior patterns “in the wild” [26]–[28]. Many of these previous efforts analyze movement-based activities such as walking, lying down, and exercising. Recognizing more complex activities such as working and shopping relies on additional information, because the movements associated with sitting in front of a television at home will be similar to the movements associated with sitting in a meeting at work. We incorporate location information to aid in detecting and distinguishing these more complex activities.

We also calculated person-specific location parameters including mean location and the top three most-frequented location regions by time of day (morning, afternoon, evening, night). For some participants, there were periods of time during transition between watches where smartwatch data were missing (mean minutes of missing readings/day = 5.59, s.d. = 5.33). We imputed the missing values using random forest regression with 100 trees. The features employed for imputation-based prediction are the previous readings from all sensors leading up to the missing time and readings for the missing sensor occurring at the same time of day (number of minutes past midnight) for the seven prior days for which readings are available. In the case of ambient sensor data from smart homes, no data were missing. These data collection environments require no actions on the part of participants. Because batteries for each sensor last for more than a year without a need for recharge, data collection did not experience any interruptions in the smart homes.

Finally, a neuropsychologist team collected self-report information for each participant and administered a battery of clinical assessments. We include a total of 129 self-report answers to health-related items and clinical parameters for analysis. Baseline self-report questions primarily assessed everyday health-related behaviors (e.g., level of engagement in physical exercise, educational community activities) based on a Likert Scale. We also collected self-report answers and performance on a cognitive n-back test [29] during data collection using an ecological momentary assessment (EMA) app. EMA self-report questions assessed in-the-moment functioning, socialization, physical and mental activity, fatigue, and mood. For the purposes of this work, these scores were averaged across the data collection period. The neurocognitive measures assessed performance across varying cognitive domains (e.g., memory, executive function, attention).

We selected 7 clinical scores as target variables for the prediction models. The target scores are summarized in Table 1. These scores were chosen because they reflect cognitive or mobility health and exhibited a sufficient variance (*>*0.1 after normalization to a [0, 1] range) among the participants. The first such score is provided by the Wechsler Test of Adult Reading (WTAR) [30]. The WTAR estimates intellectual functioning, IQ, by having participants read aloud a list of 50 words with irregular pronunciations. This assesses knowledge and reading skills that are acquired over time as the test does not rely on standard pronunciation rules. The Telephone Interview of Cognitive Status (TICS) [22] offers a cognitive screening measure that can be linked directly to the widely-used Mini-Mental State Examination (MMSE). Unlike the MMSE, the TICS can be administrated over the phone. In this test, cognitive function is assessed by having participants complete an 11-item brief screening test over the phone by completing tasks such as “count backwards by 7 from 100”. The Repeatable Battery for the Assessment of Neuropsychological Status (RBANS) [31] characterizes abnormal cognitive decline. Participants complete 12 subsets that assess the cognitive domains of attention, language, visuospatial/constructional abilities, and delayed memory. The F-A-S (FAS) [32] measures executive function. Participants are given one minute to list as many words as they can that begin with the letters “F”, “A”, or “S”. The Timed Up and Go (TUG) Test [33] is a widely-employed method of evaluating basic mobility maneuvers and has also been shown to offer indicators of cognitive and functional abilities [34].

**FIGURE 2.**
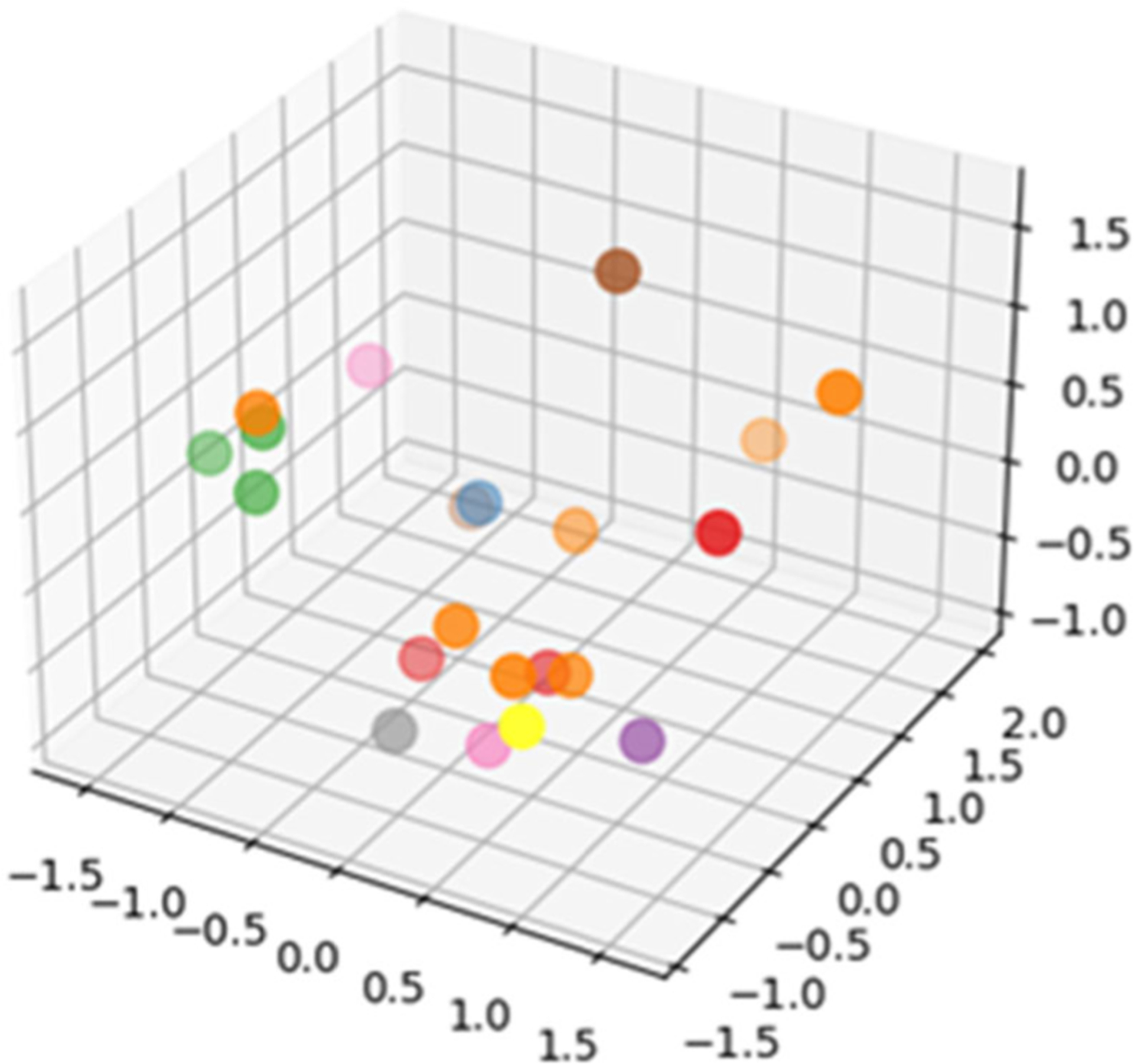
Projection of clinical variables onto a 4D space (3D geometric space and color). The project illustrates the distribution and variance of collected clinical information for the participant sample.

We also include two measures of functional abilities. These exhibit lower variance among the normalized values (0.01 for DEX, 0.07 for ADCS-ADL) among participants. However, they provide insight on how behavior patterns reflect standardized clinical assessment methods and vice versa. The first of these tests is the Dysexecutive Functioning Questionnaire (DEX) [35]. DEX asks participants to rate 20 items that are designed to assess everyday manifestations of difficulties consistent with common executive difficulties. These are emotion and personality changes, motivational changes, behavioral changes, and cognitive changes. Each item is scored on a 5-point Likert scale. The last targeted clinical score is generated by the Alzheimer’s disease Cooperative Study Instrumental protocol. In this inventory, ADCS-ADL.([36], participants rate their ability (ranging from full independence to full dependence) to complete 6 basic activities of daily living (e.g., eating) and 16 instrumental activities of daily living (e.g., cooking) over the prior 4-week period. The remaining clinical parameters are used as supplemental clinical variables in the analysis. Fig. 2 shows the distribution of the target clinical scores projected using Principal Components Analysis onto a 4D space.

### B. ACTIVITY RECOGNITION

We define a behaviorome as a set of digital behavior markers. The markers are more comprehensive if the set includes details of time spent on activities that are common and often included in clinical assessments. To include this information, we need a method that will automatically label sensor data with corresponding activity labels.

We have previously designed machine learning algorithms that label each ambient or wearable sensor reading with a corresponding activity label, referred to as Mobile-AR and Home-AR [37].^1^ Researchers have designed numerous methods for human activity recognition from smart home sensors [37]–[40] and mobile sensors [41]–[46]. However, most methods operate in controlled laboratory conditions and no previously-reported work has investigated labeling and modeling activities from both ambient and wearable sensor data. The Mobile-AR and Home-AR algorithms, in contrast, label activities in real-time for data collected in noisy, complex, realistic settings. Both algorithms employ a random forest with 100 trees to map a window of sensor readings to a corresponding activity label. The window size for ambient sensor readings is 30 readings (they do not arrive at equally-spaced time intervals) and for wearable sensor readings is 5 seconds. These window sizes have demonstrated success for activity recognition in earlier studies [25], [37], [47].

Each ambient sensor is identified based on its location and its type. Sensor locations are defined by regions of the home (bathroom, bedroom, dining room, entry, hall, kitchen, living room, office, other) and type (motion, door, temperature, light). These designators allow us to build models that generalize to new home settings without a need for floorplan information or a way to map sensor identifiers in one home to sensor identifiers in other homes. In the case of wearable data, Mobile-AR applies a Butterworth low-pass filter with a cutoff of 0.3Hz to sensor readings before extracting features to remove signal noise. The features extracted for each sensor modality are listed in Table 2, together with the activity categories that are automatically recognized for each modality.

As shown in Table 2, the learned activities are distinct for the different sensor platforms. Activity models are created from prior studies in which ground truth labels were provided for training. Activity categories were selected in those cases based on the types of activities that could be readily detected by the corresponding type of sensor. Many of the activity labels represent pre-defined activities of daily living (ADLs), while other labels are based on common behavior patterns.

The participants in the study we report here did not provide any ground truth activity labels for each smart home data or smartwatch data. As a result, we rely on pre-trained models to accurately provide activity information. Because the accuracy of the labels will impact the reliability of the extracted behaviorome, we evaluated the pre-trained models for their predictive performance on the activities listed in Table 2.

We started by performing a leave-one-house-out performance analysis for Home-AR. The evaluation is based on 94 homes containing a total of 22,602,331 sensor reading sequences with corresponding ground truth activity labels. Table 3 summarizes the performance results for Home-AR. Similarly, we evaluate Mobile-AR using leave-one-subject-out evaluation for 250 individuals who provided activity labels for a total of 1,485,438 sensor reading sequences. Table 4 summarizes the performance results for Mobile-AR. For both platforms, activity recognition performs at an f1 score of 0.85 or higher. This provides some evidence supporting inclusion of activity labels in the behaviorome that we describe in the next section.

### C. CREATING A BEHAVIOROME

Using the activity-labeled time series data, we compute and compile digital behavior markers, which become the person’s behaviorome. The set of digital behavior markers includes a set of behavior markers for each day and each hour of the day to describe observed behavior within that time period. From this information, we then additionally extract a set of overall behavior markers.^2^

The daily, hourly, and overall behavior markers are summarized in Table 5. As this table indicates, statistical summaries are gathered for each separate (daily, hourly) time period as well as for the entire data collection time period. In the case of smartwatch data, additional sensor data were available that were not labeled with the primary activity categories listed in Table 2. For some earlier studies, participants were asked to provide any appropriate activity label. Many of these diverse labels only appeared for a few participants, represented specialized activities, or indicated a person’s location context rather than an activity category. However, these extra labels can be valuable in building a behaviorome. Therefore, we train additional one-class random forest classifiers to recognize whether a person’s current context belongs to one of these categories. For all learning tasks, the class distributions are imbalanced. To address this challenge, we weight each training point. Weights are inversely proportionate to the corresponding class relative frequency in the training data. As shown in Table 5, the set of behavior markers includes time spent in one of these specialized activities. The one-class categories are: airplane, art, bathe, beach, biking, bus, car, chores, church, computer, cook, dress, drink, entertainment, groom, hobby, lunch, movie, music, relax, restaurant, school, service, shop, socialize, and sport.

Furthermore, the overall behavior markers contain information about a person’s behavior routine as a whole, as observed over the entire data collection period. These include computation of a set of regularity indicators and circadian rhythm values. A regularity index is calculated using the hourly data and compares the uniformity of a person’s schedule. This index is calculated for a within-week measure to compare days with a week, within-weekday measure to specifically determine the regularity of a Monday-through-Friday schedule, and between weeks to determine if there is a longer-term uniformity of schedule. The indices are calculated based on total acceleration, total rotation, and total distance traveled values. To calculate a regularity index, the data are first scaled to the range [−0.5, 0.5]. As introduced by Wang *et al.* [5], the regularity index comparing days a and b is defined as:

(1) $$RI_{a,b}\sum_{t=1}^{T}\frac{x_{t}^{a}x_{t}^{b}}{T}$$

For our analysis, T = 24 hours and $x_{t}^{a}$ represents the scaled value for hour *t* of day *a*.

Finally, we compute values that reflect the circadian rhythm of a person’s routine. The computation is applied only to the markers extracted for hourly time periods and is computed based on total acceleration, total rotation, and distanced traveled during the corresponding time period. To quantify circadian rhythm strength, we estimate a power spectral density using a periodogram. Based on Fourier analysis, the spectral energy of each frequency can be calculated using Equation 2.

(2) $$R={\left(\frac{2}{N}\sum_{i=1}^{N}x_{i}\text{cos}\left(\frac{2\pi jt_{i}}{N}\right)\right)}^{2}+{\left(\frac{2}{N}\sum_{i=1}^{N}x_{i}\text{sin}\left(\frac{2\pi jt_{i}}{N}\right)\right)}^{2}$$

In Equation 2, N represents the number of samples in the time series, *x*_*i*_ represents the value of the measured variable at time *i*, and the spectral energy is computed for a particular frequency, *j*. We calculate the spectral energy for a range of possible frequencies, resulting in a periodogram. This periodogram generates values to a set of possible cycle lengths. The computed circadian rhythm is then represented by the normalized periodogram-derived value for a 24-hour cycle [48].

**FIGURE 3.**
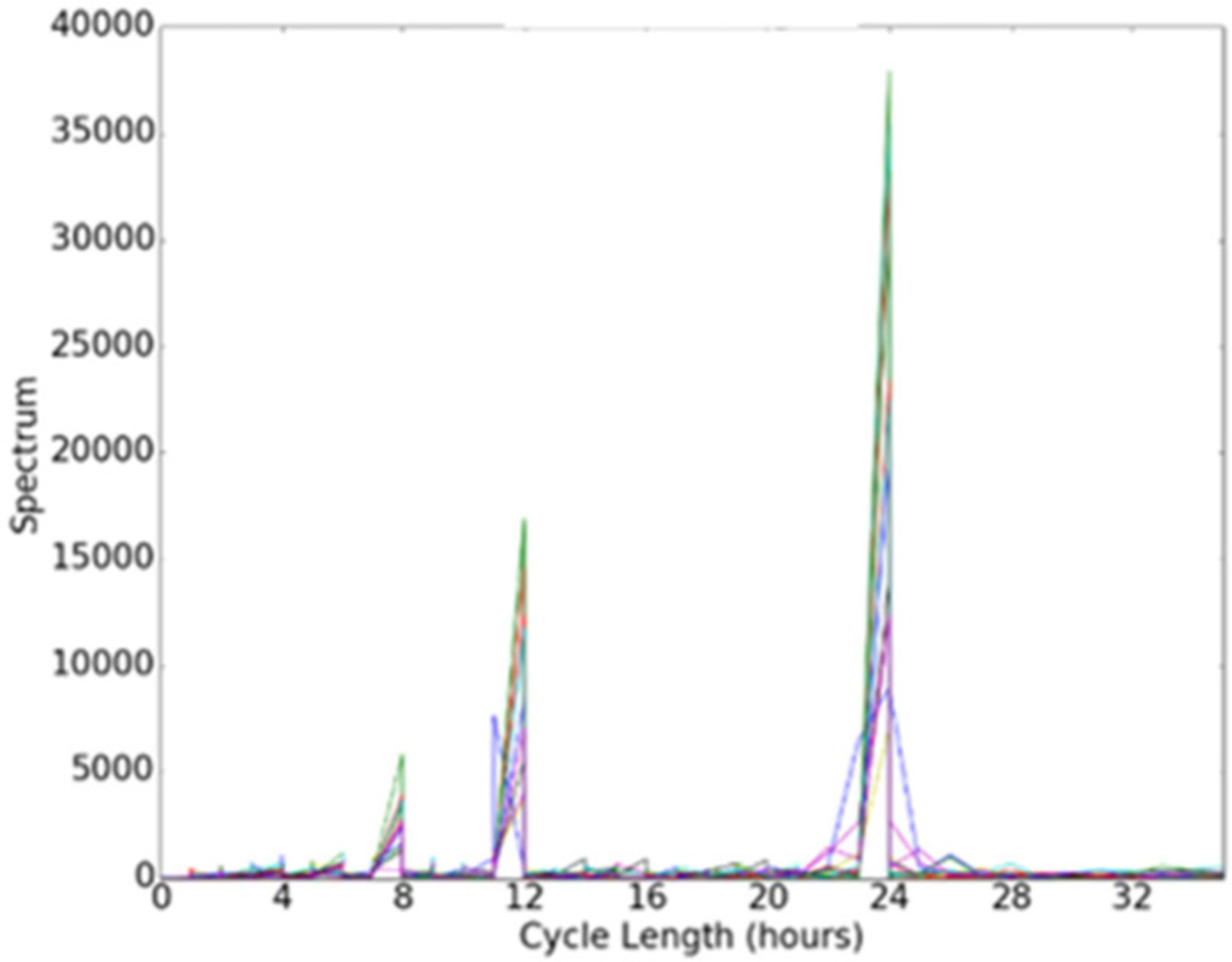
Circadian cycle strengths gleaned using a periodogram. The graph plots strengths of cycle sizes ranging from 0 to 34 hours.

Fig. 3 illustrates a periodogram that was constructed using sensor data collected from one smart home (the graph contains separate plots for each week of data collection). In the case of this home, the sensor data most strongly support a 24-hour cycle length. In the next section, we will examine whether a behaviorome constructed from these features can predict the clinical state of participants in our study.

### D. JOINT INFERENCE OF CLINICAL SCORES

The goal of this work is to predict clinical scores from a sensor-derived behaviorome. We hypothesize that these digital behavior markers provide an indication of a person’s health status and thus offer the ability to predict clinical assessment scores. We further postulate that the relationship between multiple assessment measures that are typically collected can boost the predictive performance, by leveraging the relationship.

**FIGURE 4.**
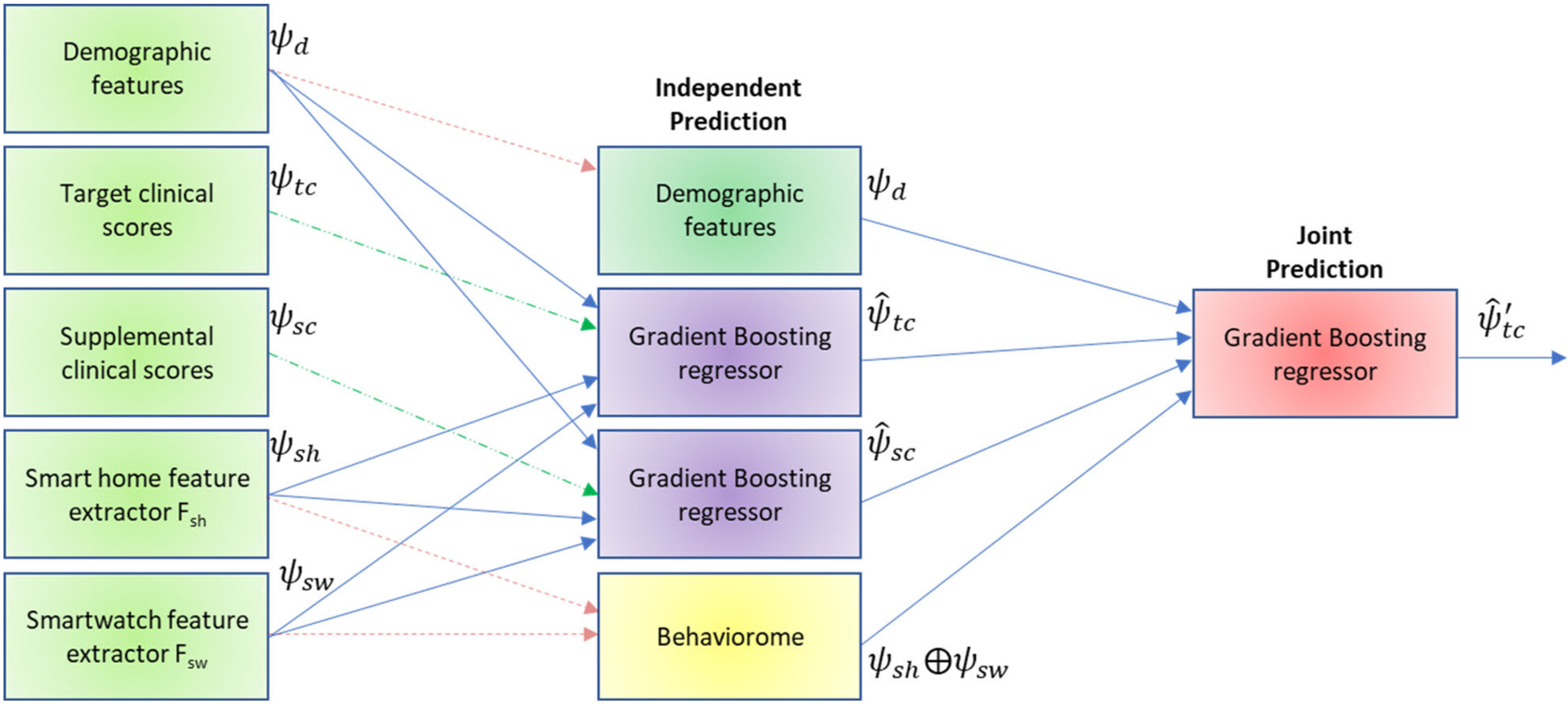
The joint inference process. Demographic variables and digital behavior markers are initially used to predict values for the target clinical scores and supplemental clinical scores, in an independent inference step. During joint inference, the predicted clinical scores for all variables except i are combined with demographic features and the behaviorome to predict the value of clinical score i. Dotted lines indicate variables that are used for both rounds. Dashed lines indicate features that are used as ground truth only during training.

Fig. 4 illustrates the joint inference process. The intuition is that demographic and sensor information is used to infer values for each clinical parameter. In a second step, demographic and sensor information is combined with the inferred clinical parameters values (from step one) to predict values for each clinical parameter. If our hypothesis is valid, the inferred values from step two (joint inference) will result in greater predictive accuracy than the inferred values from step one (independent inference).

The independent predictor is our baseline clinical score predictor. As the name suggests, this predictor ignores the relational structure of the scores, and makes predictions using only information from the demographics and behaviorome. Here, the term “demographic” is broadly used to indicate the population sector and the health background of the person (e.g., substance abuse, high blood pressure). No clinical variables are used to predict values for other clinical variables.

More formally, the independent predictor is trained as follows. For each subject, we collect demographic features *ψ*_*d*_, extract the digital behavior features *ψ*_*sh*_ and *ψ*_*sw*_ listed in Table 5, and gather ground truth scores for the target clinical scores *ψ*_*tc*_ listed in Table 1 as well as supplemental clinical scores *ψ*_*sc*_ described in Section IIIA. We train an independent regressor Π_*i*_ for each target and supplemental clinical score *i*. To do this, we collect the combined features as input *x*_*i*_ and the ground truth predictions as output *y*_*i*_. We feed the aggregate set of input/output pairs, ${\left\{x_{i},y_{i}\right\}}_{i=1}^{N}$ as training examples to a regression learner that seeks to minimize loss function L. For this problem, the non-negative loss function $L(x,\hat{y})\in {ℜ}^{+}$ is the loss associated with labeling a particular input $x\in {ℜ}^{+}$ as output $\hat{y}\in {ℜ}^{I}$ when the true output is $y\in {ℜ}^{I}$ (normalized mean absolute error).

Our inference goal is to return a function/predictor whose outputs have low expected loss, or correspondingly yield high predictive performance. Random forest performs well for complex problems, such as Home-AR and Mobile-AR. However, the clinical score prediction problem exhibits even higher dimensionality. The independent predictor utilizes 1,038 features (86 demographic features and 952 behavior features). The joint predictor utilizes these 1,038 features plus 7 target clinical scores and 129 supplemental clinical scores. For very high-dimensional spaces, gradient boosting has demonstrated consistent success [49]. Using gradient boosting with 100 estimators and a learning rate of 0.1, multiple training iterations are performed during which additional regression trees are added to an ensemble. Each added tree reduces the least squares loss from the previous iteration. We incorporate gradient boosting with regression trees for the independent and joint prediction steps of the process shown in Fig. 4.

During the joint prediction step, we employ joint predictors to improve predictive accuracy. These joint predictors are pseudo-independent in the sense that each regressor predicts the output for a single clinical score but has independent-predictor outputs from all of the other regressors available as part of the input feature vector to make a more-informed prediction. The feature vector to predict clinical score *i* now consists of demographic features *ψ*_*d*_, behaviorome features *ψ*_*sh*_ and *ψ*_*sw*_, inferred supplemental clinical scores ${\hat{\psi }}_{sc}$, and inferred target clinical scores ${\hat{\psi }}_{tc}$ for all variables other than i. Joint prediction offers advantages when the variables being predicted are related – one score may be easier to predict than another, and its inferred value can improve prediction for the related variable.

## IV. EXPERIMENTAL RESULTS

We evaluate the ability of the machine learning regression algorithm to predict clinical scores from the extracted behaviorome by computing Pearson’s correlation between the predicted and actual values. We compare the results using smart home behavior markers, smartwatch behavior markers, and a fusion of the two. Additionally, we compare the results generated by independent predictors with those generated by joint predictions. All results are generated from a leave-one-subject-out validation. Results are summarized in Table 6.

Correlations ranging from *r* = 0.019 (TICS, joint prediction based on smart home behavior markers) to *r* = 0.962 (RBANS, joint prediction based on smartwatch features) result from the proposed prediction method. The highest-performing prediction strategy for each clinical score is a joint inference method. In fact, the average correlation over all behavior marker combinations for independent predictors is a small correlation (*r* = 0.298) while the average for joint predictors is a moderate correlation (*r* = 0.601).

The results provide evidence that digital behavior markers from both smart home sensors and smartwatch sensors are predictive of clinical scores. The sensor modality that provides the largest predictive performance varies by category. Interestingly, a fusion of markers from both the smart home and the smartwatch does not consistently yield the highest predictive performance. While gradient boosting performs better than a random forest regressor and a regression tree classifier (which average *r* = 0.592 and *r* = 0.499, respectively, for joint prediction based on a fusion of behavior markers), the learner still appears to struggle with the high-dimensional space. Dimensionality reduction techniques can be further explored in future work, although reducing the dimensionality with PCA (number of components = 21) reduces the predictive performance to *r* = 0.259.

## V. CONCLUSION

In this study, we assess the ability of multi-modal sensor data to provide digital behavior markers that are predictive of clinical health states. To do this, we describe a method of collecting sensor data, automatically labeling the data with activity labels, and extracting features that form a person’s behaviorome. We built a behaviorome for each person in our study from a combination of smart home and smartwatch data. Using a gradient boosting regression algorithm, the method predicts clinical scores with moderate or high correlation for WTAR, RBANS, FAS, TUG. These scores include measures of cognitive and mobility-based health. The model also predicts scores with moderate correlation for DEX and ADLC, which are self-report measures of independent functioning. We further provide evidence that performing joint prediction for a large set of clinically-related variables boosts predictive performance.

Limitations of this work include the small sample size of 21 healthy older adults. Future studies will involve participants exhibiting a range of health conditions to examine the ability to further differentiate between diagnosis groups. Continued analysis may also shed light on the types of sensor modalities that are most predictive of specific clinical scores. We will also adapt the approach described here to track and predict changes in health status over time.

## Figures and Tables

**TABLE 1.** Targeted clinical scores.

Measure	Construct Assessed
Wechsler Test of Adult Reading (WTAR)	Premorbid verbal intellectual abilities
Telephone Interview of Cognitive Status (TICS)	Global cognitive status
Repeatable Battery of Neuropsychological Status (RBANS)	General neurocognitive status
F-A-S Test (FAS)	Executive friction
Timed Up and Go Test (TUG)	Functional mobility
Dysexecutive questionnaire (DEX)	Self-reported everyday executive dysfunction
Alzheimer’s disease Cooperative Study Activities of Daily Living Inventory (ADCS-ADL)	Self-reported everyday activity ability and functional independence

**TABLE 2.** Extracted features for one window of sensor data.

Ambient Sensors	Mobile Sensors
time of day (hour, seconds past midnight), day of the week	max, min, sum, mean, median, mean/median abs value, standard deviation, mean/median abs deviation of sensor values, moments, mean and variance of
window duration	Fourier Transformed sensor values
time elapsed since previous sensor reading	coefficient of variation, skewness, kurtosis, signal energy, log signal energy, power
dominant (most common) sensor in current / previous window	sensor value autocorrelation
last sensor / location to generate reading, location of last motion sensor	correlations between axes for multidimensional sensors
window complexity (entropy of numbers of readings for each sensor) number of changes in location	absolute value of successive values, time between peaks heading change rate, stop rate, overall trajectory
number of sensors generating readings	location type
number of readings for each sensor	normalized distance / direction from person’s mean location, distance traveled
time elapsed for each sensor since previous reading	location within most-frequented locations by time of day
***Activities:*** *Bed-toilet transition, Cook, Eat, Enter home, Leave home, Personal hygiene, Relax, Sleep, Wash dishes, Work*	***Activities:*** *Chores, Eat, Entertainment, Errands, Exercise, Hobby, Hygiene, Relax, School, Sleep, Travel, Work*

**TABLE 3.** Home-AR activity recognition results based on leave-one-home-out testing. Performance metrics are average precision, recall, and f1-score.

Precision	Recall	FI Score
0.86	0.85	0.85

**TABLE 4.** Mobile-AR activity recognition results based on leave-one-subject-out testing. Performance metrics are average precision, recall, and f1-score.

Precision	Recall	FI Score
0.94	0.87	0.86

**TABLE 5.** Summary of Digital Behavior Markers that comprise a person’s behaviorome. Markers are created separately for smart home data and smartwatch data, then fused into one behaviorome. In the case of both smart home and smartwatch data, markers are defined for each day, each hour, and overall.

Modality	Time Period	Markers	Modality	Time Period	Markers
	*day*	number of sensor readings number of distinct activities performed number of distinct locations visited time (seconds) spent on each activity time (seconds) spent at each location time of day (seconds past midnight) for first occurrence of each activity time of day (seconds past midnight) for first visit to each location		*day*	total acceleration total rotation number of missing readings total distance traveled time (seconds) spent on each one-class activity and each primary activity time spent at each location type time of day (seconds past midnight) for first occurrence of each primary activity time of day (seconds past midnight) for first visit to each location type
	*hour*	number of sensor readings number of distinct activities performed number of distinct locations visited time spent on each activity time spent at each location		*hour*	total acceleration total rotation number of missing readings time spent on each one-class activity time spent on each primary activity time spent at each location type
**home**	*overall*	statistics for daily behavior markers: mean, median, standard deviation, max, min, zero/mean crossings, interquartile range, skewness, kurtosis, signal energy statistics for hourly behavior markers: mean, median, standard deviation, max, min, zero/mean crossings, interquartile range, skewness, kurtosis, signal energy regularity index based on hourly values for number of sensor readings: within weeks, within weekdays, between weeks regularity index based on hourly values for number of activities performed: within weeks, within weekdays, between weeks regularity index based on hourly values for number of locations visited: within weeks, within weekdays, between weeks circadian rhythm: sensor reading count, number of activities, locations visited	**watch**	*overall*	statistics for daily behavior markers: mean, median, standard deviation, max, min, zero/mean crossings, interquartile range, skewness, kurtosis, signal energy statistics for hourly behavior markers: mean, median, standard deviation, max, min, zero/mean crossings, interquartile range, skewness, kurtosis, signal energy regularity index of total acceleration: within weeks, within weekdays, between weeks regularity index of total rotation: within weeks, within weekdays, between weeks regularity index of total distance: within weeks, within weekdays, between weeks circadian rhythm: total acceleration, total rotation, total distance

**TABLE 6.** Correlation between predicted and actual clinical scores. Results are reported using smart home behavior markers, smartwatch behavior markers, and a fusion of markers from both sensor modalities. Results are reported for each target clinical score based on independent prediction (I) and joint prediction (J).

Measure	SmartHome-I	Smartwatch-I	Fusion-I	SmartHome-J	Smartwatch-J	Fusion-J
WTAR	0.151	0.323	0.236	0.856*	0.879*	0.848*
RBANS	0.337	0.324	0.358	0.928*	0.962*	0.932*
TICS	0.180	0.358	0.354	0.019	0.035	**0.188**
FAS	0.238	0.278	0.498	0.806*	0.708*	0.764*
TUG	0.301	0.245	0.353	0.227	**0.492**	0.278
DEX	0.617	0.202	0.063	0.866*	0.881*	0.451*
ADLC	0.279	0.249	0.303	0.437*	0.391	0.252
Average	0.301	0.283	0.309	0.591	**0.621**	0.590
	Average of independent predictions = 0.298			Average of joint predictions = 0.601		

* The result is significant at p<.05.

## Footnotes

1 Code for Mobile-AR and Home-AR is available at https://github.com/WSU-CASAS/AL and https://github.com/WSU-CASAS/AL-Smarthome.

2 Code to extract digital behavior markers is available at https://github.com/WSU-CASAS/DM.
